# A meaning-centered spiritual care training program for hospice palliative care teams in South Korea: development and preliminary evaluation

**DOI:** 10.1186/s12904-021-00718-1

**Published:** 2021-02-09

**Authors:** Kyung-Ah Kang, Shin-Jeong Kim, Do-Bong Kim, Myung-Hee Park, Soo-Jin Yoon, Sung-Eun Choi, Young-Sim Choi, Su-Jin Koh

**Affiliations:** 1 College of Nursing, Sahmyook University, Seoul, Republic of Korea; 2 School of Nursing, Hallym University, 39 Hallymdaehak-gil, Chuncheon, Gangwon-do 24252 Republic of Korea; 3 Holistic Healing Institute of Sam Medical Center, Gunpo, Republic of Korea; 4 Hospice & Palliative Center, Seoul St. Mary’s Hospital, Seoul, Republic of Korea; 5 Dongbaek St. Luke Hospice, Gyeonggi-do, Republic of Korea; 6 Hospice Care Center of the Regional Cancer Center, Chungnam University Hospital, Daejeon, Republic of Korea; 7 Department of Nursing, Chungnam National University Hospital, Daejeon, Republic of Korea; 8 Department of Hematology and Oncology, Ulsan University Hospital, University of Ulsan College of Medicine, Ulsan, Republic of Korea

**Keywords:** Hospice, Palliative care, Program development, Spirituality

## Abstract

**Background:**

Spirituality is a fundamental, intrinsic aspect of human beings and should be a core component of quality palliative care. There is an urgent need to train hospice palliative care teams (HPCTs) to enhance their ability to provide spiritual care. This study aimed to develop and evaluate a meaning-centered, spiritual care training program (McSCTP) for HPCTs (McSCTP-HPCTs).

**Methods:**

The modules’ content was informed by Viktor Frankl’s meaning-centered logotherapy with its emphasis on spiritual resources, as well as the spiritual care model of the Interprofessional Spiritual Care Education Curriculum (ISPEC). Following development, we conducted a pilot test with four nurses. We used the results to inform the final program, which we tested in an intervention involving 13 members of HPCTs. We took measurements using self-administered questionnaires at three points before and after the intervention. Using descriptive statistics, the Mann-Whitney U test, and the Kruskal-Wallis test, we analyzed the participants’ demographic and career-related characteristics, as well as the degree of variance between three outcome variables: compassion fatigue (CF), spiritual care competencies (SCCs), and spiritual care therapeutics (SCT).

**Results:**

We divided the McSCTP-HPCTs into five modules. Module I: The HPCTs’ SCC evaluation, understanding the major concepts of spiritual care and logotherapy; Modules II-IV: Meaning-centered interventions (MCIs) related to spiritual needs (existential, relational, and transcendental/religious); Module V: The process of meaning-centered spiritual care. The preliminary evaluation revealed significant differences in all three outcome variables at the posttest point (CF, *p* = 0.037; SCCs, *p* = 0.005; SCT, *p* = 0.002). At the four-week follow-up test point, we only found statistical significance with the SCCs (*p* = 0.006).

**Conclusions:**

The McSCTP-HPCTs is suitable for use in clinical settings and provides evidence for assessing the SCCs of HPCTs.

**Supplementary Information:**

The online version contains supplementary material available at 10.1186/s12904-021-00718-1.

## Background

Across the world, interest in spiritual care in hospice palliative care (HPC) is increasing. HPC is a professional medical service provided by multidisciplinary teams comprising doctors, nurses, social workers, clergy, and volunteers. The aim of HPC is to relieve the physical, psychological, social, and spiritual suffering of patients with life-threatening illnesses and to improve their quality of life (QoL), as well as that of their family caregivers [1]. Spiritual support is typically related to greater patient well-being, happiness, hope, and gratitude [2]. Hence, spiritual well-being may protect one against despair at the end of life, and spiritual care is a fundamental component of quality palliative care [2–5]. Zollfrank et al. [2] pointed out that inadequate healthcare provider training is a significant barrier in patient care. According to an Interprofessional Spiritual Care Education Curriculum (ISPEC) report [6], the spiritual well-being of patients and their family caregivers is a major factor influencing healthcare outcomes such as QoL, positive coping, satisfaction with caring, and decision-making at the end of life [7, 8].

Hospice palliative care team (HPCT) nurses are specialists who take care of terminally ill patients 24 h a day; they are increasingly required to initiate discussions with these patients and their family caregivers concerning spirituality as the essence of their existence [1]. Spirituality researchers widely regard spirituality as a universal phenomenon, and people’s metaphysical beliefs are linked closely to their lives [9]. Understanding that humans are spiritual beings (whether they are religious or not) may be one of the strongest predictors for HPCT members in providing spiritual care for patients with life-threatening illnesses [10]. There is an urgent need for training to enhance the ability of HPCT members to satisfy patients’ spiritual care needs. Hence, in order to provide meaning-centered spiritual care focused on spirituality—to take care of one of the most crucial needs of human existence—HPCTs need systematic, educational training.

Previous studies have shown that HPCTs often face challenges regarding spiritual care. As such, they are unable to satisfy patients’ spiritual care needs. Because spiritual care has been confused with religious care, responsibility for it is placed in the hands of clergy [11, 12]. A group intervention study was conducted in the United States [13, 14] to improve HPCT nurses’ job satisfaction and QoL; likewise, a study that developed a spiritual care training protocol for oncology nurses as a comprehensive component of spiritual care was conducted in China [10]. In addition, a spiritual care training program, Clinical Pastoral Education for Healthcare Providers (CPE-HP), was created by Zollfrank et al. [2] to address healthcare providers’ ability to provide spiritual or religious care as part of healthcare. These three studies share the similarity of targeting the capacity of healthcare professionals (including nurses) to provide spiritual care. The current study differs from these investigations in that we propose a spiritual care training program that is meaning-centered, which is based on the core principles of Victor Frankl’s logotherapy, and an approach that has not been previously taken.

Since 2018, the scope of HPC recipients in South Korea has expanded to include patients other than those with terminal cancer, resulting in the need for more systematic care services and quality management [3, 15]. However, the HPC services provided in South Korea still focus on physical symptom management, and no systematic training programs have yet been implemented for the spiritual well-being of terminally ill patients. Moreover, there is no specified spiritual care training curriculum for HPCTs. In order to promote the QoL of patients with life-threatening illnesses, spiritual care interventions grounded in human spirituality need to be carried out. In addition, to establish spiritual care as a core component of HPC and quality control service not limited to religious support, the education and training of HPCT members should be given priority. This study aimed to produce and assess a spiritual care training program for HPCTs using Victor Frankl’s meaning-centered logotherapy approach to address the resources of spirituality. From here on, we will refer to the training program as the meaning-centered, spiritual care training program for hospice palliative care teams (McSCTP-HPCTs).

## Methods

### Study design

This is a methodological study employing a one-group, pretest-posttest design.

### Theoretical Foundation

**Fig. 1:**
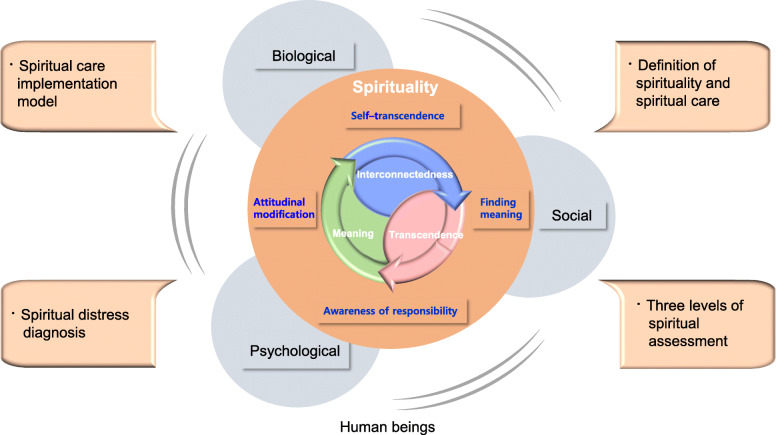
The study's conceptual framework

We created the McSCTP-HPCTs by incorporating the spiritual care guidelines formulated by ISPEC [6], as well as concepts from Viktor Frankl’s logotherapy—conceived of through his experiences in concentration camps in World War II—and we relied on meaning-centered theory to focus on and enhance the resources of spirituality (Fig. 1).

“Spirituality” refers to a dynamic, innate aspect of humanity that has a vital influence on the status of one’s body and mind [8]. A systematic description of spirituality by Lasair [9] defined it as “a person’s…body, mind, spirit, and culture bound together in a unifying metaphysical vision and experienced in time, in and through [one’s] pursuit of the good in life.” Spirituality and meaning-centered care are intimately connected in healthcare settings.

The main attributes of spirituality are meaning, interconnectedness, and transcendence [16–18]. That patients’ attitude, in the terminal stage of their illness shifts, from “pain” to “meaning” (such as the meaning of suffering, life, and death) confirms that the attributes of spirituality are linked to meaning in life. In addition, 12 primary spiritual issues (e.g., despair/hopelessness, grief/loss, guilt/shame, reconciliation, isolation etc.) suggested by the National Consensus Project for Quality Palliative Care in the United States are tied to the nature of spirituality [18]. Therefore, spiritual care should emphasize recognizing and responding to the needs of the human spirit, including aspects of spirituality through compassionate relationships [19]. ISPEC is a spiritual care training program that aims to improve spiritual care for patients, and to help healthcare providers feel confident in their ability to attend to patients’ and families’ spiritual needs. ISPEC includes an Interprofessional Special Care Model to boost the quality of spiritual care in the field of HPC. The model addresses the need for a multidisciplinary, team approach that encompasses caring for those in spiritual distress, three levels of spiritual assessment (spiritual screening, history-taking, and assessment), a compassionate presence, and communication about spiritual issues with a treatment plan [6].

Viktor Frankl described the spiritual dimension of human beings as a “healthy core” or “the defiant power of [the] human spirit” that affects one’s body and mind. In addition, the will to ascribe meaning to things is a motivating force to overcome inevitable pain and to live actively [20]. Frankl invented “logotherapy,” a theoretical system and psychotherapeutic intervention that advocates using spiritual resources to overcome unavoidable suffering. The chief assumption of logotherapy is that awareness of responsibility (being responsible for one’s own existence), finding meaning (as the motivational, driving force for relieving suffering), and self-transcendence (dedication to something beyond oneself) within an authentic encounter comprise the essence of human existence. Recovery from suffering and spiritual well-being can be achieved through attitudinal modification toward optimism in situations where pain is inescapable [20–22].

### Procedure

**Fig. 2:**
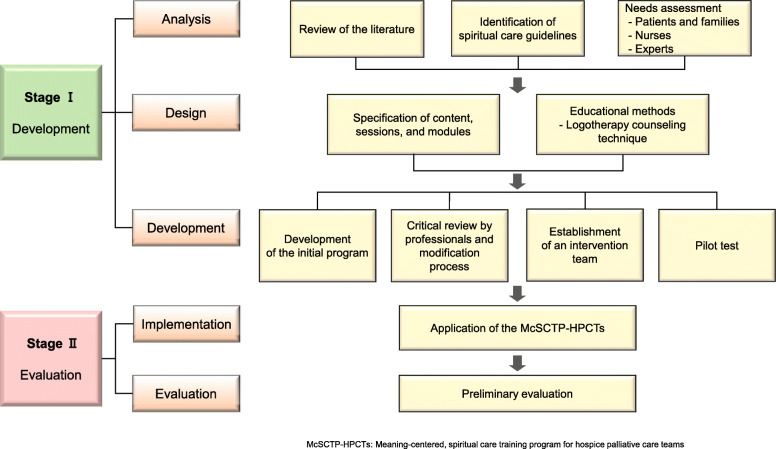
The process of this study

Figure 2 outlines the flow of the development process for the McSCTP-HPCTs, which lasted from March 2017 to April 2019. The preliminary evaluation took place from May to July 2019. We employed the ADDIE (analysis-design-development-implementation-evaluation) model of Seels and Richey for the developmental process [23] (Fig. 2).

#### Stage I: development

##### Analysis

- **Review of the literature.** From the earliest available subscription date to May 2017, we searched the literature for studies that have applied meaning-centered intervention (MCI) to patients with advanced and life-threatening diseases, as well as their caregivers. We examined the content of MCI through a systematic review [24] and two meta-analyses [25, 26]. The common purpose of MCI, identified through the analysis, is to improve spiritual well-being by finding meaning in life, even in painful situations, including incurable ailments. Regarding the major content of interventions, we found it to be comprised of the essential aspects of human existence (the meaning of life, the will to ascribe meaning to things, freedom of will, choice and responsibility, and self-transcendence), in addition to how to find meaning (creativity, experience, and attitude). Besides MCI studies—which were designed to prevent burnout among, and provide support for, nurses who provide palliative care [13, 14]—only one study on spiritual care training protocols has been conducted relating to the general educational content of spiritual care training for oncology nurses [10]. To the best of our knowledge, no McSCTP for HPCTs has been created thus far. Based on previous research, we developed the McSCTP-HPCTs to help patients find meaning in life through their own strengths, creativity, positive experiences, and attitude modification via the four key theoretical concepts proposed by logotherapy: (1) finding meaning, (2) attitudinal modification, (3) awareness of responsibility, and (4) self-transcendence.
- **The identification of spiritual care guidelines.** After searching for protocols or guidelines on spiritual care, we identified the ISPEC guidelines [6], elaborated by the National Consensus Project as an evidence-based training program for multidisciplinary teams [18]; they include specific models regarding the process of spiritual care. Therefore, the ISPEC guidelines provide an appropriate template for creating a training program suitable for South Korean culture.
- **Needs assessment.** We carried out a needs assessment, as follows. First, we identified the spiritual care needs of patients with life-threatening illnesses (and their families) who were admitted to HPC institutions in South Korea [27]. Among their spiritual care needs, the desire for love and connection, finding meaning, and hope and peace were higher than religious beliefs. As a result, we understand that spirituality (rather than religion) is a universal, intrinsic aspect of being human. Second, we administered a survey to 282 nurses working at HPC institutions (*n* = 282) on the meaning of spiritual care and their capacity for spiritual care. In response to the open-ended question “What do you think special care is?” 33.7% recognized spiritual care as “Helping prepare for a dignified death, including religious support.” [28]. A survey performed using the Spiritual Care Competency Scale (SCCS) [28] showed that the lowest-scored SCCS items were “assessment and evaluation of special care” and “professionalization and impacting the quality of special care.” Finally, we gathered opinions on spiritual care needs from a panel made up of seven experts on HPC practice, education, and officials responsible for hospice policy. The analysis process underscored the need for the McSCTP-HPCTs to be formed with due regard for the attributes of spirituality.

##### Design

- **Specification of the content, modules, and sessions.** The major content of the McSCTP-HPCTs—composed through an analysis of previous research—are the SCC evaluation of an HPCT, the concepts of spiritual care and logotherapy, and meaning-centered care linked to the three attributes of spirituality (meaning, interconnectedness, and transcendence). The program consists of five modules, for a total of 20 h.
- **Educational methods.** As primary educational methods for MCI, we applied logotherapy counseling techniques, with logo-analysis and Socratic dialogue as the main approaches. We used the Medicine Chest and Appealing Technique as complementary methods. Logo-analysis [29] is the process of discovering potential spiritual resources in one’s spirit and scrutinizing them to find meaning and purpose in life. The specific sub-processes are as follows: (1) self-evaluation; (2) acting as if …; (3) establishing an encounter; and (4) finding value in creativity, experience, attitude, and commitment (Table 1). Socratic dialogue is a way of helping people recognize the latent “logohints” in their minds through an authentic conversation with a counselor. The Medicine Chest helps patients see that there is a healthy core (the defiant power of the human spirit) in their spiritual dimension. The Appealing Technique is a self-trained meditation method that consists of positive content to reinforce the use of one’s spiritual resources.

**Table 1:** Meaning-centered spiritual care training program for hospice palliative care teams (McSCTP-HPCT)

⦁ Goal: We developed the meaning-centered, spiritual care training program (McSCTP) to promote the spiritual well-being of patients by hospice palliative care teams (HPCTs), who take care of patients with life-threatening ailments. The McSCTP is premised on humans’ spiritual attributes. ⦁ Caring principle based on McSCTP: HPCTs act as assistants to help patients with life-threatening illnesses to find their own meaning.					
	Topic	Objectives	Contents	Workbook	Methods
**Module I**	Evaluation of the spiritual care competencies (SCCs) of HPCTs and understanding the logotherapy concepts	• Identify the SCCs of HPCT members • Understand major concepts of spiritual care • Understand major concepts of logo therapy • Apply meaning-centered intervention (MCI) to oneself	• Self-evaluation of SCCs (compassion, compassion fatigue [CF], and SCCs) • Major concepts of spiritual care • Major concepts of logotherapy	• Evaluation of self-assessment regarding compassion, CF, and SCCs • Identify the case-based attributes of spirituality, spiritual needs, spiritual issues, spiritual resources /communication practices • Meaning-based perspective training with real cases • The practice of MCI for HPCTs	• Self-evaluation • Lecture • Discussion • Case study • Presentation
**Module II**	Meaning-centered care related to existential needs	• Understand the meaning-centered care process related to existential needs • Identify spiritual needs/issues/resources with real cases • Implement meaning-centered care related to existential needs	• The process of meaning-centered care related to existential needs (Sp 1) • Meaning-centered care (Sp 2)	• Identification of spiritual needs/issues/resources based on real cases • Implement meaning-centered care	• Lecture • Discussion • Case study • Practice: Meaning-centered counseling techniques • Presentation
**Module III**	Meaning-centered care linked to relational needs	• Understand the meaning-centered care process linked to relational needs • Identify spiritual needs/issues/ resources with real cases Implement meaning-centered care tied to relational needs	• The process of meaning-centered care linked to relational needs (Sp 1) • Meaning-centered care (Sp 2)	• Identification of spiritual needs/issues/resources based on real cases • Implement meaning-centered care	• Lecture • Discussion • Case study • Practice: Meaning-centered counseling techniques • Presentation
**Module IV**	Meaning-centered care related to transcendental/ religious needs	• Understand the meaning-centered care process related to transcendental/religious needs • Identify spiritual needs/issues/ resources with real cases • Implement meaning-centered care related to transcendental/religious needs • If a patient has a religious need, refer him/her to the clergy member he/she wants	• The process of meaning-centered care related to transcendental/religious needs (Sp 1) • Meaning-centered care (Sp 2)	• Identification of spiritual needs/issues/resources based on real cases • Implement meaning-centered care	• Lecture • Discussion • Case study • Practice: Meaning-centered counseling techniques • Presentation
**Module V**	Meaning-based care implementation model and caring process for spiritual well-being	• Understand the meaning-centered spiritual care model for the spiritual well-being of patients with life-threatening illnesses • Identify the implementation process of meaning-centered spiritual care for the spiritual well-being of patients with life-threatening illnesses	• Spiritual care implementation model • Spiritual care decision pathway • The principles of spiritual care • Assessment of spiritual needs and spiritual resources • Meaning-centered spiritual care process based on spirituality (Sp 1)	• Spiritual needs assessment based on a meaning-centered perspective	• Lecture • Discussion • Practice

*HPCT* Hospice palliative care team; *McSCTP-HPCTs* Meaning-centered Spiritual Care Training Program for HPCTs; *Sp* Supplementary file

##### Development

- **Developing an initial program**. To ensure effective outcomes for both patients and healthcare professionals, the program had to address both the importance of spiritual care (based on the attributes of spirituality) and the HPC provider’s level of compassion [8, 30–33]. These issues were reflected in the evaluation of compassion fatigue (CF) and the spiritual care competencies (SCCs) of HPCTs. The initial program encompassed the spirituality of the ISPEC guidelines, the meaning and standard of spirituality care, a spiritual assessment, and diagnosis based on the three attributes of spirituality, as well as basic concepts of spirituality implementation. To facilitate the efficient progress of education, we organized the McSCTP-HPCTs as a group intervention; this entailed a mix of didactic presentations, case sharing, and experiential exercises using logotherapeutic counseling approaches including logo-analysis, Socratic dialogue, group discussions with reflection, and home exercises.
- **Critical review by professionals and the modification process**. Most prior studies that have applied MCI to patients with an advanced or terminal illness or in situations of unavoidable suffering were designed as group interventions, with eight sessions lasting 90–120 min per session with lectures, discussions, and reading and self-reflection as individual tasks [24–26]. Two studies that have applied MCI to improve job satisfaction and QoL among palliative care nurses were designed with four sessions of group intervention lasting 120–180 min per session [13, 14]. The teaching methods in these two investigations involved didactic presentations, discussions, experiential exercises, and home exercises similar to those of the McSCTP-HPCTs in the present study. The educational methods of these previous investigations were planned around five sessions of 240 min each, plus group intervention. At a workshop with spiritual care experts in the HPC field, we agreed that five modules, 5 h per week, for 4 weeks, with a total of 20 h of training, would be appropriate for the educational component of the McSCTP-HPCTs. In addition, we agreed that to strengthen case-oriented education, we would narrow down the 12 spiritual issues presented in the ISPEC guidelines to nine issues suitable for South Korean culture. The McSCTP-HPCTs is based on humans’ universal spiritual attributes. Further, we modified the three levels of spiritual assessment to be appropriate for the South Korean context. We agreed that religious needs expressed by the subject should be referred to clergy.
- **Establishing an intervention team**. To ensure consistency in education, the first author of this study and one of the coauthors—who is an expert (a trained chaplain) in the field of HPC—were designated as an educator and facilitator, respectively.
- **Pilot test**. To check the suitability of the McSCTP-HPCTs, four nurses working in the tumor and HPC fields tested the problem and satisfaction level of the procedure focused on progress, as well as the content validity. Each expert checked the content validity using a 4-point Likert scale (1 = *not relevant*, 2 = *somewhat relevant*, 3 = *quite relevant*, and 4 = *highly relevant*). In this study, the content validity index was above 80% for all 10 items tested. We employed these results to complete the final McSCTP-HPCTs.

#### Stage II: evaluation

##### Implementation

- **Participants.** The participants for the preliminary evaluation were members of HPCTs who work at a nationally administered hospice care institution. The inclusion criterion was that they must have been working in an HPC unit or center for more than 5 years. Initially, 15 people participated, but two dropped out, leaving a total of 13 (eight nurses, two social workers, and three individuals from related professions).
- **Intervention procedure.** Two educators, who acted as facilitators for lectures and discussions, presented the McSCTP-HPCTs at four weekly training sessions (for a total of 5 h per week, 20 h total). At each session, they gave a lecture after briefly introducing the learning objectives and content. After the lecture, they facilitated group talks (with 3–4 participants per group) based on real cases presented in the workbook. The focus of these discussions was on the lecture content’s practical applicability based on the participants’ field experiences. Afterward, the participants shared their opinions to reflect on the session’s learning content. For data collection, the research assistant explained the study’s purpose and distributed the self-administered questionnaire. We took the measurements for the McSCTP-HPCTs over three time periods. We conducted the pretest measurement (Measure 1, M 1) before the McSCTP-HPCTs was presented. We performed the posttest measurement (Measure 2, M 2) after the training was completed, and we carried out the follow-up test (Measure 3, M 3) 4 weeks after the completion of the posttest by mail.

##### Evaluation

- **Measures.** We collected sociodemographic and career-related background data at M 1. We selected the three outcome variables (compassion fatigue, SCCs, and spiritual care therapeutics [SCT]) based on a recent finding that spiritual care provision is associated with HPCT members’ skills in providing spiritual care, decreased compassion satisfaction with silencing response, and burnout [33–35]. We gauged SCCs using the SCCS [28] on a 5-point Likert scale (1 = *completely disagree* to 5 = *fully agree*); it consists of 27 items employed to assess six sub-dimensions (the implementation of spiritual care, professionalization and improving the quality of spiritual care, personal support and patient counseling, communication, attitude toward the patient’s spirituality, and referrals to professionals). The Cronbach’s alpha was .94. The Spiritual Care Therapeutics Scale [34] evaluates the frequency of HPCT-provided spiritual care; it comprises 17 items, rated using a 5-point Likert scale (1 = *never*, 2 = *rarely*, 3 = *occasionally*, 4 = *often*, 5 = *very often*). The Cronbach’s alpha was .97. CF involves the silencing response experienced by HPC providers in the early stages of burnout [36]; we measured it by means of 16 items using a 5-point Likert scale (1 = *never*, 2 = *rarely*, 3 = *occasionally*, 4 = *often*, 5 = *very often*). The scale exhibits internal reliability, with an alpha coefficient of 0.85. After the translation-reverse translation process, five experts validated both SCT and CF. The content validity index was above 80% for all items.
- **Data analysis.** We analyzed the data using the Statistical Package for Social Sciences (IBM SPSS, Version 25.0). Using descriptive statistics, the Mann-Whitney U test, and the Kruskal-Wallis test, we examined participants’ demographic and career-related characteristics, as well as the degree of variance between the outcome variables. We tested the effects of the McSCTP-HPCTs using a paired t-test to determine the changes in score between the measurement points.

## Results

### Developing the McSCTP-HPCTs

We divided the McSCTP-HPCTs into five modules, described briefly below and in more detail in Table 1 (see Supplementary Tables 1 and 2). Each module encompasses learning objectives, key training content, and workbooks, and includes case-based discussions and exercises for effective, practical application.

#### Module I

This module entails the evaluation of the HPCT members’ spiritual care skills, their understanding of the major concepts of spiritual care and logotherapy, and the direct application of MCI to HPCTs. To enhance the ability of HPCT members to provide MCI, they practiced self-evaluation to find meaning in their jobs.

#### Module II

This module is made up of an MCI process that presents two spiritual issues (“despair/hopelessness” and “lack of meaning and purpose”) related to patients’ existential needs.

#### Module III

Module III contains an MCI process involving five spiritual issues (“anger at God or others,” “guilt/shame,” “grief/loss,” “abandonment by God or others/isolation,” and “reconciliation”) tied to the relational needs of patients and their families.

#### Module IV

The contents of this module are linked to transcendental/religious needs, along with two spiritual matters: “concerns about one’s relationship with a deity” and “conflicted or challenged belief systems.”

#### Module V

This final module reconstructs the process of meaning-centered spiritual care in the context of ISPEC’s Spiritual Care Implementation Model; it comprises two parts. The first one entails a meaning-centered spiritual care model, including a spiritual implementation model, decision pathways, and caring principles for spiritual well-being. The second part covers a spiritual care matrix (spiritual assessment with three levels: screening, history, and assessment/spiritual resources; and needs based on spiritual attributes, spiritual issues, and an MCI evaluation).

The workbooks for modules II, III, and IV contain practical exercises to identify spiritual needs (existential, relational, and transcendental), as well as to find ways to satisfy such needs with spiritual resources, as well as other spiritual matters grounded in actual cases.

### Evaluation

#### Participants’ background characteristics and differences in outcome variables

Table 2 outlines the participants’ characteristics. None of the items showed significant differences in the mean scores of the three outcome variables. The participants’ characteristics were homogeneous with regard to the outcome variables (CF, SCC, and SCT).

**Table 2:** Participants’ background characteristics and differences in the outcome variables (*N* = 13)

Characteristics	Categories	M (SD)/N (%)	CF		SCC		SCT	
			M (SD)	***p***	M (SD)	***p***	M (SD)	***p***
Age (years)		44.69 (9.69)	–	–	–	–	–	–
	< 39	6 (46.2)	2.26 (0.42)	.445^*^	3.12 (0.69)	.366^*^	3.07 (0.75)	.445^*^
	> 40	7 (53.8)	2.11 (0.31)		3.48 (0.24)		3.35 (0.32)	
Marital status	Not married	6 (46.2)	2.27 (0.34)	.534^*^	3.44 (0.42)	.628^*^	3.25 (0.58)	.836^*^
	Married	7 (53.8)	2.10 (0.38)		3.22 (0.59)		3.19 (0.57)	
Educational level	Undergraduate	9 (69.2)	2.09 (0.39)	.260^*^	3.33 (0.57)	.604^*^	3.25 (0.59)	.604^*^
	Graduate	4 (30.8)	2.37 (0.18)		3.29 (0.41)		3.16 (0.53)	
Religion	Have	11 (84.6)	2.13 (0.37)	.231^*^	3.31 (0.56)	.923^*^	3.26 (0.57)	.513^*^
	None	2 (15.4)	2.44 (0.09)		3.39 (0.18)		3.03 (0.54)	
Type of job	Nurse	8 (61.5)	2.34 (0.32)	.099^†^	3.38 (0.41)	.922^†^	3.26 (0.50)	.872^†^
	Social worker	2 (15.4)	2.06 (0.35)		2.93 (1.26)		2.85 (1.21)	
	Other	3 (23.1)	1.81 (0.17)		3.42 (0.15)		3.35 (0.27)	
Experience of hospice care education	Have	9 (69.2)	2.28 (0.35)	.148^*^	3.25 (0.61)	.604^*^	3.18 (0.66)	.825^*^
	None	4 (30.8)	1.94 (0.28)		3.47 (0.16)		3.31 (0.24)	
Length of clinical career (years)		153.38 ± 94.40	–		–		–	
	Under 5	2 (15.4)	1.66 (0.04)	.080^†^	3.61 (0.39)	.521^†^	3.56 (0.71)	.580^†^
	5–10	3 (23.1)	2.19 (0.22)		3.00 (0.85)		2.86 (0.76)	
	Above 10	8 (61.5)	2.31 (0.33)		3.37 (0.39)		3.27(0.45)	
Length of hospice care career (years)		84.92 (54.51)	–		–		–	
	Under 5	5 (38.5)	2.04 (0.36)	.321^†^	3.13 (0.68)	.722^†^	2.94 (0.75)	.298^†^
	5–10	5 (38.5)	2.36 (0.30)		3.44 (0.38)		3.34 (0.39)	
	Above 10	3 (23.1)	2.10 (0.45)		3.43 (0.48)		3.49 (0.28)	
Educational needs for spiritual caring	Have	12 (92.3)	–		–		–	
	None	1 (7.7)	–		–		–	

*CF* Compassion fatigue

*SCC* Spiritual care competencies

*SCT* Spiritual care therapeutics

^*:^ Mann-Whitney U test

†^:^ Kruskal-Wallis test

#### Comparison of changes in the outcome variables

Table 3 displays the differences in the mean score by measurement points. Measure I (M1–M2) revealed significant differences in all three outcome variables (CF, *p* = 0.037; SCC, *p* = 0.005; SCT, *p* = 0.002). There was no significant difference among the sub-dimensions of SCCS, except for the communication sub-dimension (SCCS-C, *p* = 0.252). In Measure II (M1–M3), we only found statistical significance for the SCC (*p* = 0.006). We did not detect any significant differences for CF (*p* = 0.123) or SCT (*p* = 0.464).

**Table 3:** Changes in CF, SCCs, and SCT from baseline through follow-up (*N* = 13)

Variables (items)	Measure 1 (M1-M2)		Measure 2 (M1-M3)	
	Diff (SD)	t (***p***)	Diff (SD)	t (***p***)
CF (16)	0.21 (0.32)	2.35 (.037)	0.16 (0.35)	1.66 (.123)
SCC (27)	−0.48 (0.50)	−3.50 (.005)	−0.45 (0.48)	−3.38 (.006)
SCC-A (6)	−0.60 (0.64)	−3.40 (.005)	−0.54 (0.67)	−2.90 (.013)
SCC-PI (6)	−0.54 (0.70)	−2.77 (.017)	−0.53 (0.61)	−3.12 (.009)
SCC-PP (6)	− 0.44 (0.66)	−2.39 (.034)	− 0.38 (0.70)	−1.98 (.072)
SCC-R (3)	−0.49 (0.50)	−3.50 (.004)	− 0.44 (0.60)	−2.62 (.022)
SCC-At (4)	−0.38 (0.54)	− 2.59 (.024)	− 0.38 (0.44)	− 3.15 (.008)
SCC-C (2)	− 0.27 (0.81)	−1.20 (.252)	− 0.31 (0.69)	− 1.60 (.136)
SCT (17)	− 0.35 (0.31)	−4.04 (.002)	− 0.09 (0.41)	− 0.76 (.464)

M1: pretest

M2: posttest

M3: follow-up (after 4 weeks)

*CF* Compassion fatigue

*SCCs* Spiritual care competencies

*SCC-A*: assessment of the implementation of spiritual care

*SCC-PI* Professionalization and improving the quality of spiritual care

*SCC-PP* Personal support and patient counseling

*SCC-R* Referral to a professional

*SCC-At* Attitude toward the patient’s spirituality

*SCC-C* Communication

*SCT* Spiritual care therapeutics

## Discussion

### Developing the McSCTP-HPCTs

We elaborated the McSCTP-HPCTs to help HPCT members maximize patients’ spiritual resources; it relates to human spirituality, rather than religious aspects [20–22]. The theoretical background is rooted in ISPEC’s Spiritual Care Implementation Model, as well as the logotherapy approach, which is a meaning-centered approach (versus a pathos-centered one) [20, 21]. In previous research, meaning in life has been reported as a stable, intrapersonal resource that can be used to maintain the spiritual well-being of patients with chronic or life-threatening illnesses [37, 38].

Regarding the main features of the McSCTP-HPCTs, they unfolded as follows. First, the medical personnel’s own spirituality and compassion skills were dealt with for spiritual care. Their spirituality affects healthcare outcomes, including QoL [18]. Compassion is a spiritual practice, a way of being, a way of serving others, and an act of love. Thus, spirituality is intrinsically linked to compassion [8, 39]. HPCT members’ compassion and spiritual care skills were assessed before providing spiritual care; compassion training was also emphasized. Further, the self-reflection process enabled the HPCT members to discover meaning in their own profession as part of their training prior to spiritual care [7]. Zollfrank et al. [2] also stressed the importance of nurses’ professional meaning and commitment to spiritual care.

Second, the McSCTP-HPCTs is tied to spiritual needs with expressions, spiritual issues, and MCI based on the attributes of spirituality. In studies investigating the spiritual needs of patients who require hospice care and their families in South Korean culture [27, 40], spiritual needs—grounded in the spirituality of patients and their families—were all high in the order of interconnectedness, meaning, and religious demands. Further, in a study on perceptions of spiritual care among patients with life-threatening cancer, primary family caregivers and hospice nurses [41] demonstrated that spiritual care is commonly seen as relating to having the opportunity for internal reflection, finding meaning, encouraging hope, and listening to and being with patients. These results underscore the need to increase the understanding of spiritual care based on aspects of universal human spirituality, as well as the need for meaning-oriented (versus purely religion-centered) spiritual care. We designed the McSCTP-HPCTs to meet the existential needs of terminally ill patients and to promote their spiritual well-being, which is a critical outcome and core component of quality in oncology and palliative care [38].

Finally, we grounded the McSCTP-HPCTs in the concepts of spirituality presented by ISPEC, as well as an interdisciplinary approach to spiritual assessment, the implementation model, and spiritual matters. Puchalski et al. [18] underscored the importance of spiritual care in palliative care settings, and clarified who should offer spiritual care and the role of healthcare teams in administering it. To date, although some researchers have highlighted the significance of spiritual care, it has not been systematically provided, especially for patients with life-threatening conditions, due to the insufficient preparedness of HPCTs [8]. The spiritual assessment—the third stage of spiritual assessment outlined by ISPEC—includes a question about patients’ spiritual resources (see Supplementary Table 1) [30]. Questions such as this one can identify the spiritual resources shown in the Medicine Chest, one of the logotherapy counseling techniques [30]. Therefore, HPCTs must carefully pay attention to and care for their patients’ spirituality; part of HPCT members’ role is to safeguard it. Accordingly, they are able to help patients cope with their terminal illness and treatment using the defiant power of spirituality [30]. In addition, this aids clinicians in conceptualizing and planning for subsequent treatment. Moreover, we narrowed down the 12 spiritual issues depicted in the ISPEC guidelines [6] to nine issues suitable for South Korean culture, centered around meaning (“despair/hopelessness” and “lack of meaning and purpose [existential]”), interconnectedness (“anger at God or others,” “guilt/shame,” “grief/loss,” “reconciliation,” and “abandonment by God or others/isolation”), and transcendence (“concerns about one’s relationship with a deity” and “conflicted or challenged belief systems”). We excluded two spiritual topics (“religious-specific”, and “religious/spiritual struggle”) and merged two issues (“abandonment by God or others” and “isolation”) because of cultural differences based on the spiritual needs assessment [27, 28]. This suggests that the framework and content of spiritual care training should consider variations according to cultural differences, but still follow global standard guidelines [42, 43]. In addition, personnel who undergo spiritual care training are more likely to meet patients’ spiritual needs [44, 45]; by receiving such training, HPCTs can more effectively assist patients in finding meaning in life and in overcoming the spiritual suffering experienced during illness.

### Evaluation

In the preliminary evaluation, we chose three outcomes (CF, SCCs, and SCT) to gauge changes in the competence with which HPCTs provide spiritual care. We tested CF to identify the HPCTs’ own self-preparedness, and we employed the SCCS to assess their ability [28]. We harnessed the Spiritual Care Therapeutics Scale to calculate the frequency of HPCT-provided spiritual care [34]. However, in this study, for the first post-measurement, all three variables (CF, SCCs, and SCT) exhibited significant differences at M 2 compared to the pretest scores, but in the measurements after 4 weeks, only the SCCs were maintained significantly. The maintenance effect for CF and SCT may have been short-lived because it was difficult to apply the content of the McSCTP-HPCTs continuously after training, since only one or two people per institution participated. Hence, we recommended that all HPCTs at the institution participate in the McSCTP-HPCTs, and that continuous application and evaluation be established simultaneously [46].

### Clinical implications

Spiritual care education is a type of interprofessional team training in HPC settings [18, 38, 39]. We, the authors, expect that the McSCTP-HPCTs will help HPCTs to learn techniques they can use to provide effective spiritual care for their patients. Thus, the McSCTP-HPCTs may facilitate the development and improvement of HPCT members’ ability to provide spiritual care to diverse patients with life-limiting illnesses or conditions, as well as their families.

In addition, this study highlights the importance of spiritual care training, which can impact spiritual well-being in patients with life-threatening illnesses. The better we understand and are aware of HPCTs’ spirituality, as cultivated by the McSCTP, the more probable it is that the McSCTP-HPCTs could be utilized in some interventions that are explicitly oriented toward spiritual issues. The McSCTP-HPCTs can help to enhance the spirituality of patients suffering from life-threatening ailments, as well as that of their family members. Since the aims of spiritual care include easing patients’ difficulties, helping them to find meaning in life, and improving their spiritual well-being [10], the McSCTP-HPCTs can help patients to attain these goals, as well as to understand their own sense of value.

### Limitations

The limitations of this study should be acknowledged. First, the McSCTP-HPCTs is a training program to help HPCTs provide spiritual care, with a focus on meeting patients’ existential needs. We did not include communication, ethics, or religious care in the educational content. Regarding communication, we only dealt with compassion training through reflective listening; we did not include the overall concepts and domains of communication. Second, we developed the McSCTP-HPCTs with a focus on ISPEC’s inpatient Spiritual Care Implementation Model. When considering the outpatient situation, program modification and further testing are required. We employed a one-group, pretest-posttest design with a small number of participants. We are planning a follow-up study using a control group, with a sample size suitable for parametric analysis. Finally, a tool used to measure the CF of HPCTs is needed to verify objective validity for conceptualization. This tool should consist of certain themes (e.g., beliefs and attitudes around spirituality; the knowledge, ability, and frequency of spiritual care) as an early sign of CF. In addition, the SCCS is a self-report questionnaire that depends on participants’ evaluation; it needs to be tested objectively in future research.

## Conclusions

To better integrate spiritual care in clinical practice, it is necessary to create and increase the importance of spiritual care among HPCTs through effective training programs. Using the ISPEC guidelines and logotherapy, we formed a spiritual care training program for HPCTs (the McSCTP-HPCTs), comprised of five modules. The preliminary test showed that this study may be used as evidence for future research to test the effectiveness of the McSCTP-HPCTs by appraising the spiritual care skills of HPCTs.

## Supplementary Information

**Additional file 1: Supplementary Table 1.** Meaning-centered, spiritual care process founded on spirituality for HPCTs.

**Additional file 2: Supplementary Table 2.** Meaning-centered spiritual care record sheet for HPCTs.

## Data Availability

The data of this study can be obtained by any reasonable request from authors with permission of the National Research Foundation of Korea. If needed, please contact the author of this article.

## Abbreviations

CF: Compassion fatigue
HPC: Hospice palliative care
HPCT: Hospice palliative care team
ISPEC: Interprofessional spiritual care education curriculum
MCI: Meaning-centered intervention
McSCTP-HPCT: Meaning-centered spiritual care training program for hospice palliative care team
SCC: Spiritual care competencies
SCCS: Spiritual care competence scale
SCT: Spiritual care therapeutics
QoL: Quality of life
