# Correction to: A meaning-centered spiritual care training program for hospice palliative care teams in South Korea: development and preliminary evaluation

**DOI:** 10.1186/s12904-021-00760-z

**Published:** 2021-05-11

**Authors:** Kyung-Ah Kang, Shin-Jeong Kim, Do-Bong Kim, Myung-Hee Park, Soo-Jin Yoon, Sung-Eun Choi, Young-Sim Choi, Su-Jin Koh

**Affiliations:** 1 College of Nursing, Sahmyook University, Seoul, Republic of Korea; 2 School of Nursing, Hallym University, 39 Hallymdaehak-gil, Chuncheon, Gangwon-do 24252 Republic of Korea; 3 Holistic Healing Institute of Sam Medical Center, Gunpo, Republic of Korea; 4 Hospice & Palliative Center, Seoul St. Mary’s Hospital, Seoul, Republic of Korea; 5 Dongbaek St. Luke Hospice, Gyeonggi-do, Republic of Korea; 6 Hospice Care Center of the Regional Cancer Center, Chungnam University Hospital, Daejeon, Republic of Korea; 7 Department of Nursing, Chungnam National University Hospital, Daejeon, Republic of Korea; 8 Department of Hematology and Oncology, Ulsan University Hospital, University of Ulsan College of Medicine, Ulsan, Republic of Korea

**Correction to: BMC Palliative Care 20, 30 (2021)**

**https://doi.org/10.1186/s12904-021-00718-1**

Following publication of the original article [1], the authors reported that the Figures were not included in the published version.

The correct Figs. 1 and 2 and its captions have been included in this correction, and the original article has been corrected.

**Fig. 1:**
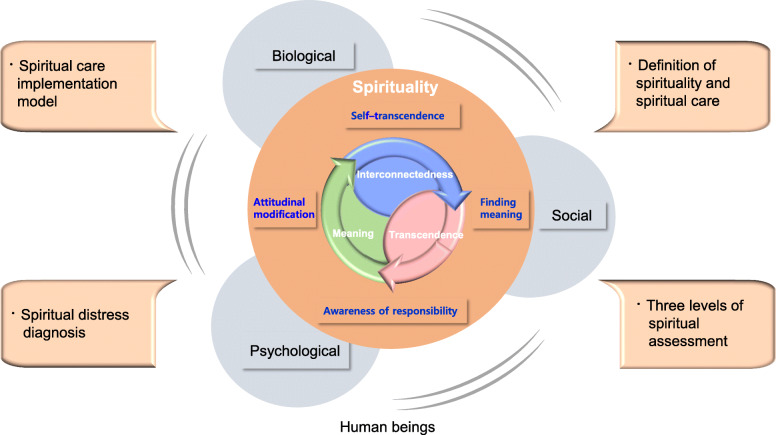
The study’s conceptual framework

**Fig. 2:**
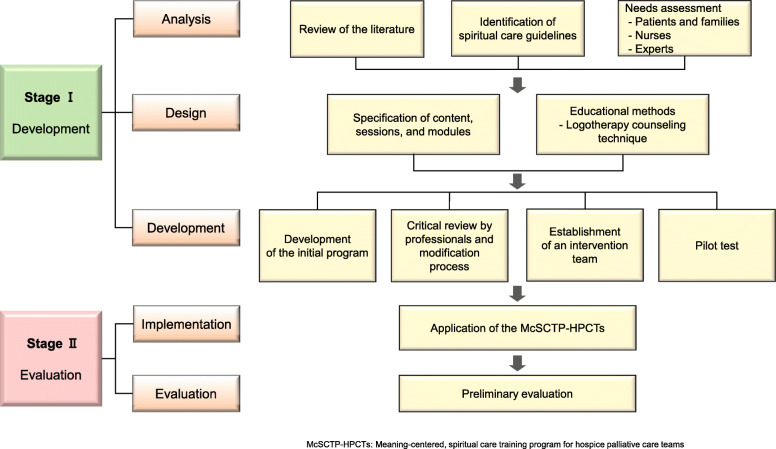
The process of this study
