# Supplementary material for: A meaning-centered spiritual care training program for hospice palliative care teams in South Korea: development and preliminary evaluation
Source: BMC Palliat Care. 2021 Feb 9;20:30. doi: 10.1186/s12904-021-00718-1 (PMC7871309; doi:10.1186/s12904-021-00718-1)
Supplement: Supplementary file 1 — Additional file 1: Supplementary Table 1. Meaning-centered, spiritual care process founded on spirituality for HPCTs. [file 12904_2021_718_MOESM1_ESM.docx]

**Supplementary Table 1** Meaning-centered, spiritual care process founded on spirituality for HPCTs

| **Attributes of spirituality** | **Spiritual needs** | **Indicators/Expressions of spiritual needs** | **Spiritual issue** | **Objectives of intervention** | **Meaning-centered intervention** | **Evaluation**  **(one item)^*^** | **Final outcome** |
| --- | --- | --- | --- | --- | --- | --- | --- |
| Meaning | Existential needs | - Loss of joy and hope - The feeling of futility in one’s life - Asking questions like “Why did this happen to me?” - A desperate attitude: “It’s not worth living.” - Apathy, a lack of concern, melancholy, and helplessness - “How can I find hope when there is none?” - The experience of losing a loved one | Despair/hopelessness | Finding hope and meaning | Sp 2 | Regain one’s motivation through hope and finding meaning | Spiritual well-being |
|  |  |  | Lack of meaning and  purpose | Finding hope and meaning |  | Find hope and  valuable meaning |  |
| Inter-connectedness | Relational needs | - Hatred and anger toward others - “I got cancer because stressed me.” - “They didn’t let me feel at ease at all; I can’t forgive them.” - “Why should I die from cancer?’ | Anger at God or others | Restoring love and relationships with others (gratitude) | Sp 2 | Restore love and relationships through gratitude | Spiritual well-being |
|  |  | - Anxious - “I can’t sleep because I'm nervous.” - “If I close my eyes, I feel like I’m going to die.” - “If I die, I’m going to hell for a lot of sins.” - Expressions of guilt - “My sins are too heavy.” - “I’m a sinner.” - A lack of self-worth - “Because of my sins, I can’t live.” | Guilt/shame | Restoring love and relationships with others (being forgiven) |  | Restore love and relationships through being forgiven |  |
|  |  | - Crying, lack of appetite, difficulty getting through each day - A gloomy appearance - Rejecting everything - Unstable - An uneasy look - Lonely appearance, expressing solitude in words - “I’m so lonely.” - “I’m alone.” - Excessive praise - A hateful act (to get attention) - Asking for attention and help - “Please pay attention to me.” - Blaming others for ignoring him/her. - Living apart from others - No visitors and does not get along with other patients. - Feels pity for oneself - “I’m such a pitiful man.” - Requests to meet the priest - Reads scripture and prays - Wants to identify the love of the Absolute | Grief/loss | Restoring love and  relationships with others  (acceptance of reality) |  | Restore love and relationships through acceptance of reality |  |
|  |  |  | Abandonment by God or others/isolation | Restoring love and  relationships with others (a sense of bonding) |  | Restore love and relationships by being connected |  |
|  |  | - A regretful look: Why did I do that at the time... I didn’t know that I hurt you... | Reconciliation | Restoring love and  relationships with  others (reconciliation) |  | Restore love and relationships through reconciliation |  |
| Transcendence | Transcendental/ Religious needs | - Fear of being alone - Obsessed with a specific person or thing - Regret over one’s own faults - “God will not abandon me.” | Concerns about one’s relationship with a deity | Restoring love and  relationships with others (hope) | Sp 2 | Restore love and relationships by finding hope | Spiritual well-being |
|  |  | - Lingering attachments and regrets about life - What happens after death? - “If there’s an afterlife, I want to go to heaven.” - “There will be a heaven without pain waiting for me.” - Thinking a miracle will happen owing to the development of new drugs. - Feeling that there is a light at the end of the tunnel. | Conflicted or challenged belief systems | Restoring love and  relationships with  others (belief in  the afterlife) |  | Restore love and relationships believing in the afterlife |  |

HPCT: hospice palliative care team

Sp: supplementary file

^*^5 point Likert scale: 1-Not at all, 2-A little, 3-Somewhat, 4-Quite a bit, 5-Very much
