# Supplementary material for: A meaning-centered spiritual care training program for hospice palliative care teams in South Korea: development and preliminary evaluation
Source: BMC Palliat Care. 2021 Feb 9;20:30. doi: 10.1186/s12904-021-00718-1 (PMC7871309; doi:10.1186/s12904-021-00718-1)
Supplement: Supplementary file 2 — Additional file 2: Supplementary Table 2. Meaning-centered spiritual care record sheet for HPCTs. [file 12904_2021_718_MOESM2_ESM.docx]

**Supplementary Table 2** Meaning-centered spiritual care record sheet for HPCTs

| **∎ Step 1. Building trust and compassion** | | | | |
| --- | --- | --- | --- | --- |
| **Specific goal** | **Preparedness** | **Meaning-centered care plan** | **Record** | |
| Build trust and compassion through a spiritual assessment and careful observation, and listening regarding the spiritual concerns of patients with life-threatening illnesses or at the end of life. | - Compassion and listening | - Relationships with patients with life-threatening illnesses (3-point scale) | 1-Bad,  2-Somewhat  3-Good | |
|  |  | - Assess (or evaluate) the CF of the HPCTs |  |  |
|  |  | - Patients’ spiritual assessment (screening, history, and assessment) |  |  |
|  |  | - Spiritual issue |  |  |
|  |  | - Objectives of intervention |  |  |
|  |  |  |  | |
| **∎ Step 2. Distancing from suffering and despair** | | | |  |
| **Specific goal** | **Preparedness** | **Meaning-centered care plan** | **Record** |  |
| Help patients to find their worth in a painful situation, and to keep negative feelings and despair (from loss) at bay. | - Changes in interests (humor, nature photography, etc.) | - Identify strengths of patients: Activity  \| ▶ Activity: My strength note \| \| \| \| \| \| \| \| \| --- \| --- \| --- \| --- \| --- \| --- \| --- \| --- \| \| My strength \| Day 1 \| Day 2 \| Day 3 \| Day 4 \| Day 5 \| Day 6 \| Day 7 \| \| 1. \|  \|  \|  \|  \|  \|  \|  \| \| 2. \|  \|  \|  \|  \|  \|  \|  \| \| 3. \|  \|  \|  \|  \|  \|  \|  \| |  |  |
| **∎ Step 3: Relaxation of tension from current difficulties** | | | | |
| **Specific goal** | **Preparedness** | **Meaning-centered care plan** | **Record** | |
| Help the patient to relieve tension experienced in painful situations | - Meditation | - The Appealing Technique leads to spiritual resources |  | |
|  |  | - Encourage terminally ill patients to read their spiritual resources and “My strengths” three times a day. |  | |

| **∎ Step 4: Searching for one’s value and potential spiritual resources** | | | |
| --- | --- | --- | --- |
| **Specific goal** | **Preparedness** | **Meaning-centered care plan** | **Record** |
| Help the patient to identify his/her own  meaning resources by  exploring the past, present and future | - Quotes from Viktor E. Frankl | - My positive resources  \| ▶ Activity 1: My life mountains  -An important life event in the past.  -The power that made you stay positive through this event.  -The effects of positivity on the suffering of an end-stage disease. \| \| --- \| \| ▶ Activity 2: My life map  -Memorable moments with happy experiences, people, and relationships \| | Spiritual resources  □ Meaning in life  □ The will to find meaning  □ Freedom of choice  □ Responsibility & response-  ability  □ Self-awareness &  discovery  □ Creativity  □ Commitment to tasks  □ Gratitude  □ Compassion and  forgiveness  □ Love (altruistic)  □ Sense of humor (optimism)  □ Awareness of mortality  □ Self-transcendence |
|  |  | - Encourage patients to build spiritual resources observed during this time. - Encourage patients to read “My strengths” more than three times a day. |  |

| **∎ Step 5: My creativity** | | | |
| --- | --- | --- | --- |
| **Specific goal** | **Preparedness** | **Meaning-centered care plan** | **Record** |
| Help patients to identify their creativity and responsibility | - Quotes from Viktor E. Frankl | - Finding my creativity: Activity  \| \| ▶ Activity: Finding my creativity \| \| \| --- \| --- \| \| 1 \| Search for my talent. \| \| 2 \| ⦁ List everything you want to do now (including goals that are impossible) based on this talent.  -For finding hope and meaning  -For restoring love and relationships \| \| 3 \| ⦁ Among the items on the list, choose one thing that is the most meaningful and realistic. \| \| 4 \| Think about what this choice means. \| \| 5 \| Write down a specific plan for the final choice. \| \| \| --- \| --- \| --- \| --- \| --- \| --- \| --- \| --- \| --- \| --- \| --- \| --- \| --- \| | Spiritual resources  □ Meaning in life  □ The will to find meaning  □ Freedom of choice  □ Responsibility & response-ability  □ Creativity  □ Commitment to tasks |
|  |  | - Encourage patients to build spiritual resources observed during this time. - Encourage patients to read “My strengths” more than three times a day. |  |

| **∎ Step 6: Reflecting on one’s experiences** | | | |
| --- | --- | --- | --- |
| **Specific goal** | **Preparedness** | **Meaning-centered care plan** | **Record** |
| Help patients understand the importance of interconnectedness (relationships) as an experiential resource of meaning | - Quotes from Viktor E. Frankl | - Activity: Finding my positive experiences  \| \| ▶ Activity: Finding my positive experiences \| \| \| --- \| --- \| \| 1 \| ⦁ Write down your experiences of joy and gratitude in your relationship with the Absolute (God), nature, and the universe.  - -One’s most precious person   \| 1. \| \| --- \| \| 2. \| \| 3. \|   - -Nature, art, travel experiences   \| 1. \| \| --- \| \| 2. \| \| 3. \|   - -The experience of gratitude   \| 1. \| \| --- \| \| 2. \| \| 3. \| \| \| 2 \| ⦁ Choose one of the most meaningful and actionable experiences on the list. \| \| 3 \| Think about the meaning of this choice. \| \| 4 \| Write down a specific practice method for your final choice. \| \| \| --- \| --- \| --- \| --- \| --- \| --- \| --- \| --- \| --- \| --- \| --- \| --- \| --- \| --- \| --- \| --- \| --- \| --- \| --- \| --- \| | Spiritual resources  □ The will to find meaning  □ Freedom of choice  □ Gratitude  □ Compassion and forgiveness  □ Love (altruistic)  □ Self-transcendence |
|  |  | - Encourage patients to build spiritual resources observed during this time - Encourage patients to read “My strengths” more than three times a day. |  |

| **∎ Step 7 Modification of attitude** | | | |
| --- | --- | --- | --- |
| **Specific goal** | **Preparedness** | **Meaning-centered care plan** | **Record** |
| Help patients identify freedom and their ability to choose a positive attitude during an unchangeable adversity (finding positivity in painful situations). | - Quotes from Viktor E. Frankl | \| - Activity: Finding my positivity  \| ▶ Activity: Finding my positivity \| \| \| --- \| --- \| \| 1 \| ⦁ List your own coping methods, current difficulties, and hidden positive aspects.  -My own coping method to overcome difficulties   \| 1. \| \| --- \| \| 2. \| \| 3. \|   -Current difficulties   \| 1. \| \| --- \| \| 2. \| \| 3. \|   -Positivity in difficulties   \| 1. \| \| --- \| \| 2. \| \| 3. \| \| \| 2 \| ⦁ Choose one thing that gives you the greatest meaning from the positivity list. \| \| 3 \| ⦁ Write down a specific plan that can maintain the positive meaning of the final choice. \| \| \| --- \| --- \| --- \| --- \| --- \| --- \| --- \| --- \| --- \| --- \| --- \| --- \| --- \| --- \| --- \| --- \| --- \| --- \| | Spiritual resources  □ The will to find meaning  □ Freedom of choice  □ Responsibility & response-  ability  □ Gratitude  □ Sense of humor (optimistic)  □ Self-transcendence |
|  |  | - Encourage patients to build spiritual resources observed during this time. - Encourage patients to read “My strengths” more than three times a day. |  |

| **∎ Step 8 Identify ultimate meaning in my life** | | | |
| --- | --- | --- | --- |
| **Specific goal** | **Preparedness** | **Meaning-centered care plan** | **Record** |
| Help patients to find their own spiritual resources and long-term meaning in life. | - Quotes from Viktor E. Frankl | - Activity: Finding the ultimate meaning in my life  \| \| ▶ Activity: Finding the ultimate meaning in my life \| \| \| --- \| --- \| \| 1 \| What does this life-threatening illness mean to me?  (What does my inner self really want, encounters with myself) \| \| 2 \| The power to make me feel alive during the rest of my life. \| \| 3 \| Newly discovered purpose and ultimate meaning in my life. \| \| 4 \| Specific plans for my purpose and ultimate meaning. \| \| \| --- \| --- \| --- \| --- \| --- \| --- \| --- \| --- \| --- \| --- \| --- \| | Spiritual resources |
|  |  |  | □ Gratitude  □ Compassion and  forgiveness  □ Love (altruistic)  □ Sense of humor (optimistic)  □ Awareness of mortality  □ Self-transcendence |
|  |  |  | □ Meaning in life  □ The will to find meaning  □ Freedom of choice  □ Responsibility & response-  ability  □ Creativity |
|  |  | - Encourage patients to build spiritual resources observed during this time. - Encourage patients to read “My strengths” more than three times a day. |  |

HPCT: hospice palliative care team; CF: compassion fatigue
